# The use of educational intervention on cleaning process in a secondary hospital

**DOI:** 10.1186/2047-2994-4-S1-P32

**Published:** 2015-06-16

**Authors:** AF Sola, FS Vasques, CCD Oliveira, ACC Pignatari

**Affiliations:** 1 Controle de infecção, São Paulo, Brazil; 2 Hodpital 9 de Julho, São Paulo, Brazil

## Introduction

Early in the twentieth century, health professionals became more concerned about the need to provide a clean environment and adherence to aseptic practices when caring for people´s health. In that setting, the use of chemical products for cleaning and disinfecting was widespread.For that reason, cleansing is a relevant issue in hospital infection control. By ensuring proper hygiene of hospital items and areas, cross-infection can be reduced.

## Objectives

This study aims to evaluate the technique and effectiveness of the environmental cleansing performed by the hospital hygiene team.

## Methods

This is a qualitative and quantitative prospective study, conducted from January to October 2014 in a 300-bed high complexity hospital, Sao Paulo, Brazil. In this study, we evaluated 2880 items in 360 observations before and after the cleaning team were trained on correct cleaning procedures. Evaluation and measurement were divided in three steps: (a) cleaning technique was monitored by using a visible signaling system with black light and by the measurement of the amount of adenosine trisfofate (ATP) detected on the surfaces. Measurements took place before and after the environmental cleansing;(b) the intervention was applied and the team was trained on correct cleaning procedures.

## Results

During the training period, measurements continued to take place with the same techniques; (c) the cleaning effectiveness was measured again after the training period. A target of 70% compliance was set by the infection control team. During the study period, the average compliance was 67.97%. Analysis before and during intervention showed 51.69% and 78.82% compliance, exceeding the set target.

## Conclusion

In order to have a biologically safe hospital environment it is necessary to work with different environmental peculiarities, establish partnership with different services of the institution and staff, and implement effective actions to control the spread of microorganisms, particularly multi-resistant ones.

## Disclosure of interest

None declared.

